# Deciphering Novel Communication Patterns in T Regulatory Cells From Very Old Adults

**DOI:** 10.1111/acel.70044

**Published:** 2025-03-18

**Authors:** Tegan McTaggart, Jing Xuan Lim, Katie J. Smith, Bronagh Heaney, David McDonald, Gillian Hulme, Rafiqul Hussain, Jonathan Coxhead, Derek A. Mann, Avan A. Sayer, Antoneta Granic, Shoba Amarnath

**Affiliations:** ^1^ Biosciences Institute Newcastle University Newcastle Upon Tyne UK; ^2^ NIHR Biomedical Research Centre Newcastle Upon Tyne UK; ^3^ Centre for Cancer Research The Medical School, Newcastle University Newcastle Upon Tyne UK; ^4^ AGE Research Group Translational and Clinical Research Institute Newcastle Upon Tyne UK

**Keywords:** 85, aging, CD8^+^CD56^+^T cells, the very old, TIGIT, Tregs

## Abstract

Regulatory T cells (Tregs) are important in maintaining tolerance and are key players in immunity. In aging, increased Treg function along with low‐grade inflammation has been reported. This dichotomy of enhanced Treg function along with inflammation highlights the importance of understanding Treg biology and communication patterns in the very old. In this proof‐of‐concept study, we demonstrate that aged Tregs (85 years) do not significantly communicate with CD4^+^ and CD8^+^ T effectors when compared with healthy < 66‐year‐olds. Of note was the enhanced communication of aged Tregs with CD3^+^CD8^+^CD56^+^CD161^−^ NK‐like T‐cell populations, which are important in antitumor and chronic viral diseases in older individuals. We found that in turn this population of killer‐like T cells showed diminished cytotoxic characteristics, and killer receptor expression. Taken together, our proof‐of‐concept study delineates the biology of Tregs and identifies previously undefined communication patterns in the very old.

## Graphical abstract

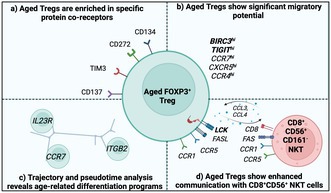

## 1 Introduction

Aging is an inevitable process that results in the progressive dysregulation of the immune system. Immunological aging is associated with declined immunity (immunosenescence) and chronic non‐specific inflammation (inflammaging), which can contribute toward several age‐related diseases (Cavanagh et al. 2012; Hohensinner et al. 2011). The phenotype and function of the aging immune system have been extensively studied, with several reports highlighting an increase in effector memory T cells, resulting in the disruption of immune homeostasis (Goronzy et al. 2013). The accumulation of conventional T cells, along with an increase in inflammatory cues within the aging immune system, suggests a breakdown in immune regulatory pathways during aging (Cavanagh et al. 2011).

Regulatory T cells (Tregs) are an important population that play a key role in maintaining immune regulation. Tregs are characterized by the expression of the IL2R (CD25), IL7R (CD127), and the master transcription factor FOXP3 (Abbas et al. 2013). The frequency and function of Tregs have been extensively studied both in aged murine models and in studies of older adults, resulting in contradictory findings (Jagger et al. 2014; Palatella et al. 2022). In humans, FOXP3 can also be induced by T‐cell receptor activation (Walker et al. 2003; Amarnath et al. 2007), thereby highlighting a major challenge in studying this key regulatory population in age. Furthermore, functional Treg suppressor assays are also unreliable due to the expression of FOXP3 and CD25 in effector T cells, thereby skewing the functional understanding of Tregs in humans. Hence, in healthy aging, some reports demonstrate an increase in Treg numbers (Gregg et al. 2005) with an enhanced suppressor function (Sun et al. 2012; Vukmanovic‐Stejic et al. 2013; Lages et al. 2008) whereas in other studies, Treg function has been shown to be diminished with age (Guo et al. 2020; Tsaknaridis et al. 2003). Mechanistically, murine studies have shown that an increase in Treg senescence is associated with elevated reactive oxygen species (ROS) production, which impairs Treg function with age (Guo et al. 2020). In humans, an accumulation of Tregs in older adults has been noted in several studies, with an increase in memory phenotype, which is not attributed to increased proliferation but to a reduction in the pro‐apoptotic molecule Bim (Chougnet et al. 2011; Raynor et al. 2015). Hence, in older adults, Tregs accumulate and are anti‐apoptotic.

The role of co‐inhibitory receptors in Treg function has been well established in age. The expression of conventional co‐receptors such as CTLA4 and GITR is unchanged with age, while ICOS has been implicated in aged Treg accumulation (Raynor et al. 2015). The expression pattern of other canonical co‐inhibitory receptors and their respective function in aged Tregs has also been described (Cavanagh et al. 2011). Taken together, a significant proportion of studies have addressed the basic biology of human Tregs in age but do not address the complex interactions of Treg with other immune cells within an aged immune system. Given the significant caveats associated with functional Treg suppressor assays, one avenue to discern Treg function would be to understand the crosstalk of Tregs with conventional and unconventional immune cells in age. This crosstalk can provide a detailed understanding of Treg function in age without the confounding factors of TCR‐driven FOXP3‐expressing T‐cell contamination in functional assays.

To unravel the complex age‐associated Treg crosstalk with other immune cells in the periphery, we sought to investigate the major ligand‐receptor interactions of Tregs in older adults using BD Rhapsody single‐cell analysis. Combining protein and gene expression, we elucidate the biology of aged Tregs in very old individuals, using a cohort of 85‐year‐olds, and identify key regulatory crosstalk of Tregs with a killer‐like CD8^+^ T‐cell subset that exists in human peripheral blood. This killer‐like CD8^+^ T‐cell population has been implicated as a key population in protecting hosts from cancer and chronic viral diseases (Almehmadi et al. 2014; Kawarabayashi et al. 2000; Koh et al. 2023). In this study, we aim to understand the immune system in 85‐year‐old individuals to close the knowledge gap in immune biology in this age group, utilizing < 66‐year‐old individuals as the control cohort. Using these two cohorts, we generate a roadmap on immune regulatory cells in 85‐year‐old individuals. Hence, our study has endeavored to explore pathways that are operational within Tregs in very old adults, which can predict immunity against cancer and viral diseases.

## 2 Results

### 2.1 BD Rhapsody Single‐Cell Sequencing Enables Characterization of Immune Cell Populations in Aging

**FIGURE 1:**
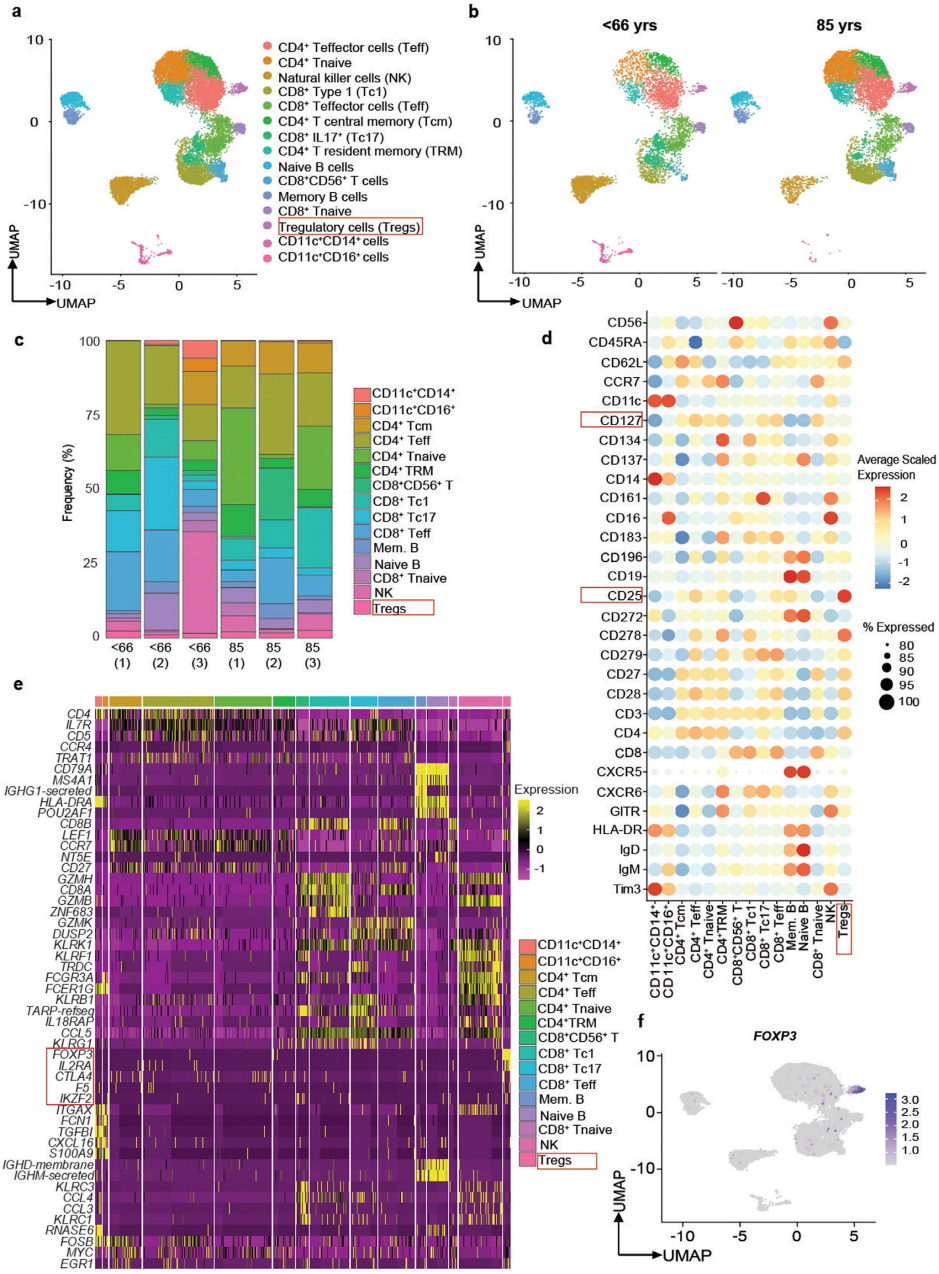
Single‐Cell Analysis of Peripheral Blood Immune Cells in 85‐year‐old individuals. PBMCs from < 66‐year‐old controls and 85‐year‐old individuals were subjected to BD Rhapsody single‐cell analysis using 30 AbSeq antibodies and a 399 immune gene panel. Cell clusters were visualized using a UMAP (a), divided into < 66‐year‐old and 85‐year‐old samples (b), and the frequency of each immune cell cluster is shown (c). AbSeq antibodies were used for defining the various immune cell populations (d), and then unbiased clustering was performed showing the top five genes per cluster (e). UMAP visualization showing transcript expression of *FOXP3* (f) is shown. Data shown are from *n* = 3 samples for < 66 and 85‐year‐old individuals, with 6009 and 8103 cells analyzed, respectively. Tcm, T central memory cells; Teff, T effectors; TRM, resident memory T cells; Tc1, type 1 CD8^+^ T cells; Tc17, IL17 producing CD8^+^ T cells; NK, natural killer cells; Tregs, T regulatory cells; Mem. B, memory B cells.

Most immune aging studies are restricted to adults aged less than 80 years with minimal information available on those aged 85 years and older (Karagiannis et al. 2023), particularly with regard to the role of Tregs. The MASS_Pilot Study (Dodds et al. 2018) provides the opportunity to characterize immune composition in deep phenotyped women and men aged 85 years. In order to determine age‐specific defects in the peripheral immune system, using previously published protocols by our group (Lim et al. 2023), we performed a BD Rhapsody single‐cell analysis using 30 AbSeq antibodies and a 399 transcript gene panel on 14,112 peripheral blood mononuclear cells (PBMC) from 85‐year (8103 cells) and < 66‐year‐old individuals (6009 cells). Using a UMAP visualization plot, we found that the two cohorts clustered in a similar fashion (Figure 1a,b) and the frequency of the Treg cluster in individual samples was of the diversity expected from human immune cells (Figure 1c). To define immune cell populations, AbSeq antibodies were used, enabling a comprehensive classification of the various immune clusters present in the samples via protein expression. We found several immune cell clusters determined by protein expression (Figure 1d), and this correlated to an unbiased transcript clustering heatmap (Figure 1e , Table S1). In this study, we focused on elucidating the phenotype and immunobiology of FOXP3^+^ Tregs in older adults. Using UMAP visualization, we noted a clear FOXP3^+^ Treg population, which was distinctly identifiable from the single‐cell analysis of peripheral blood (Figure 1d–f). Further, using AbSeq antibodies, we confirmed the FOXP3 cluster expressed *FOXP3* mRNA, CD25 protein, and exhibited diminished CD127 protein expression (Liu et al. 2006; Seddiki et al. 2006) (Figure 1d). No change in Treg frequency was noted between < 66‐ and 85‐year‐old individuals (S1a). Additionally, co‐receptor expression within this dataset demonstrated that canonical co‐receptors such as CTLA4 and TIGIT were also detected within the Treg cluster (Figure 1d, Figure S1b–e).

### 2.2 Aged Tregs Show Significant Migratory Potential and Skewed Co‐Receptor Expression

**FIGURE 2:**
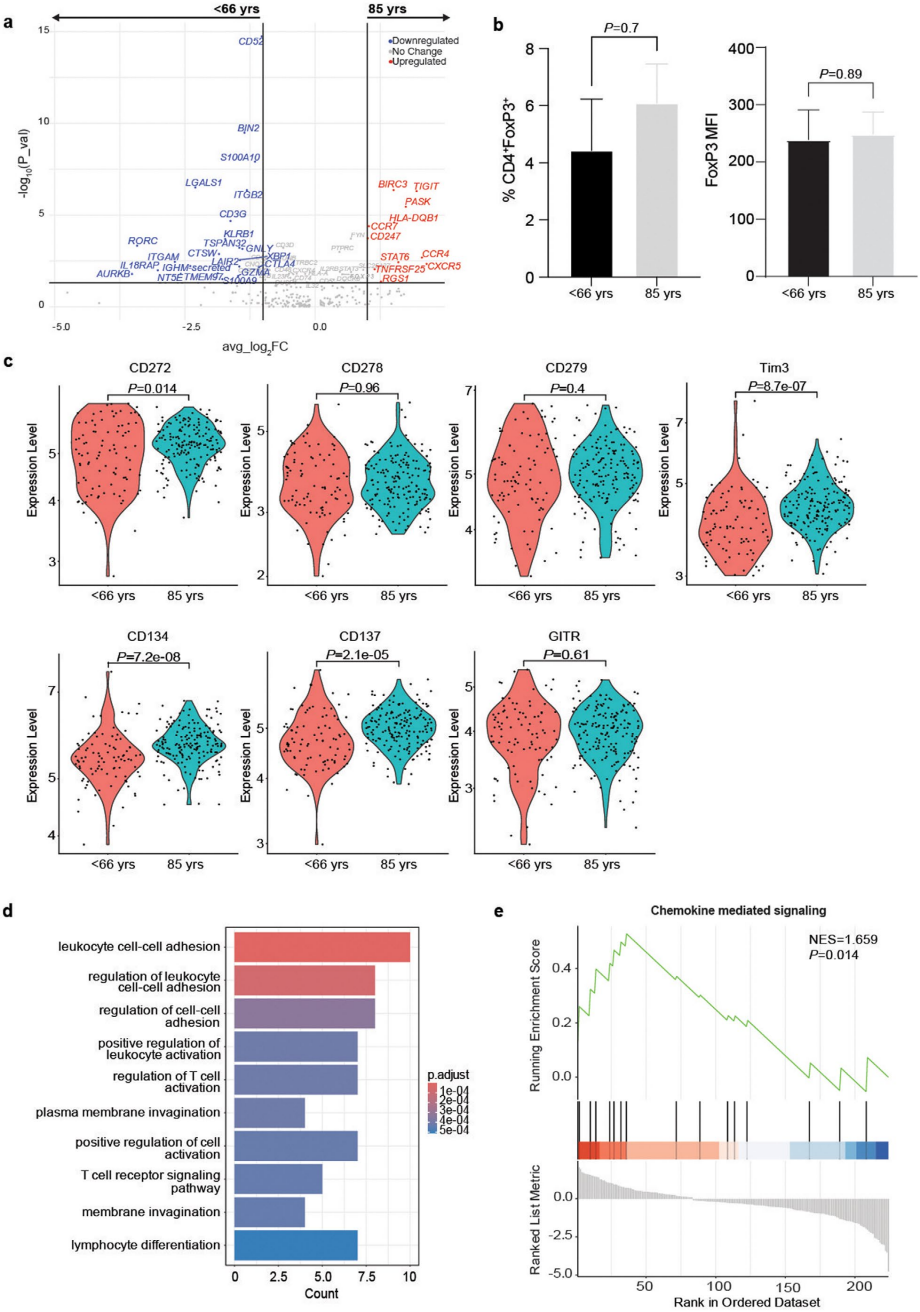
Characterization of 85‐year‐old Tregs versus < 66‐year‐old Tregs. Differential gene expression analysis was performed in Tregs from the < 66 and 85 cohorts, visualized using a volcano plot (a), with upregulated and downregulated genes in 85‐year‐old Tregs defined by log_2_ fold‐change > 1 or < −1 and a *p* value < 0.05. Frequency and mean fluorescence intensity (MFI) of FoxP3 protein in < 66‐ and 85‐year‐old Tregs measured by flow cytometry (b). Conventional and unconventional protein co‐receptor expression (detected using AbSeq) on < 66‐ and 85‐year‐old Tregs (c) is shown using violin plots. GO pathway analysis of 85 Tregs (d), along with increased chemokine‐mediated signaling shown by gene set enrichment analysis (e). Statistical analysis for flow cytometry data were done using an unpaired Student's *t*‐test. Statistical analysis for violin plots was done using the Wilcoxon rank sum test.

We performed differential gene expression analysis of Tregs from the < 66 and 85 cohort, visualized using a volcano plot. This analysis revealed that *FOXP3* is not substantially increased in the 85‐year‐old cohort (Figure 2a, Table S2). We did observe a significant increase in the co‐inhibitory receptor transcript *TIGIT*, chemokines including *CCR4, CXCR5*, and *CCR7*, characteristic of Tregs, Follicular regulatory T cells (TFR), and homing chemokines, along with an increase in the anti‐apoptosis gene (*BIRC3*) (Figure 2a).

Using flow cytometry, we next analyzed protein expression of FoxP3 to further validate mRNA analysis. We found that there was no significant difference in Treg frequency between the < 66‐ and 85‐year‐old cohorts, nor was there a change in protein expression (Figure 2b). The quantitative protein expression of conventional and unconventional co‐receptors (CD272/BTLA) on aged Tregs was also analyzed using AbSeq antibodies. An increase in CD272, Tim3, CD134 (OX40), and CD137 (41‐BB) was noted, while no changes were observed in GITR, ICOS (CD278), and PD‐1 (CD279) expression (Figure 2c) within the Treg cluster. It should be noted that although PD‐1 was not significantly increased, a population of PD1^+^TIGIT^−^ Tregs exists in the very old (population 12, 85 cohort), based on flow cytometry analysis (Figure S2a). Hence, our AbSeq protein data suggest that aged Tregs are enriched in specific co‐receptors. We then determined the GO pathways enriched in old Tregs and found that they were enriched in crosstalk with other immune cells (Figure 2d, Table S3). Gene set enrichment analysis further revealed a significant enrichment for chemokine‐mediated signaling pathways (Figure 2e, Table S4).

### 2.3 Aged Tregs Exhibit Several Differentiation Programs Compared to Control Tregs

We next performed trajectory and pseudotime analysis in SeqGeq software, using the Monocle Plugin. Pseudotime and single‐cell trajectory analysis allow for the unbiased characterization of cell states within single‐cell datasets by identifying how far a cell has progressed through a biological process, transitioning from one state to another. The trajectory analysis identifies cellular decisions, with each branching node indicating how cells choose between various potential end states (Trapnell et al. 2014).

**FIGURE 3:**
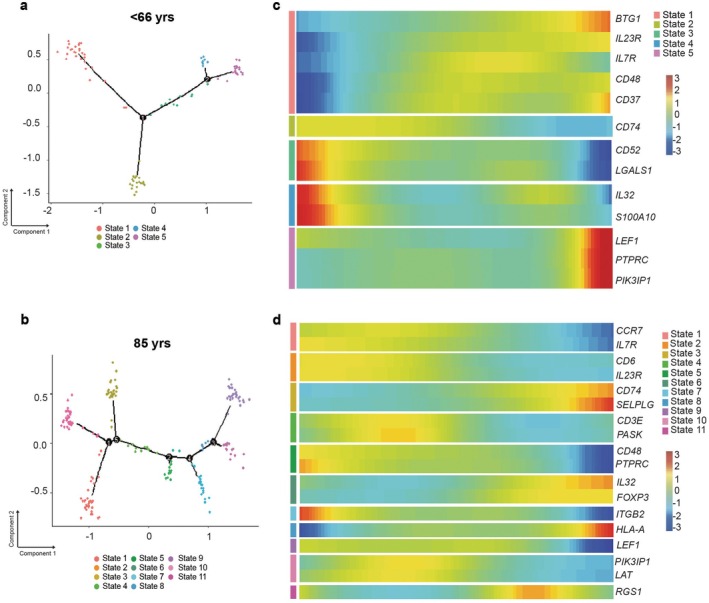
Monocle and Pseudotime analysis demonstrate an upregulation of Treg differentiation states in 85‐year‐old individuals. The Monocle plugin on SeqGeq software was used to perform pseudotime and trajectory analysis. In < 66 Tregs, two points of divergence with five states of differentiation (a) were observed, shown as a branched trajectory graph. In 85 Tregs, five points of divergence were observed with eleven differentiation states (b). Each dot represents a single Treg cell, each branch represents a state, and a numbered point represents the divergence of a state. Heatmap visualization of branch‐dependent genes across pseudotime for each state defined in < 66‐year‐old Tregs (c) and 85‐year‐old Tregs (d). In the heatmap, the increase in gene expression is denoted as red, with decrease or absence of gene expression as blue, denoting the trajectory of gene expression in different states within Tregs. AbSeq was excluded from the parameter list.

In this analysis, we found that while Tregs in the < 66‐year‐old cohort possessed 5 different states, in the 85‐year‐old cohort, 11 distinct Treg states were found (Figure 3a,b). These different states in the very old may reconcile some of the contradictory data in the literature regarding the varied function of aged Tregs. Within these 11 states, we found states expressing genes such as *ITGB2*, which was restricted to 85‐year‐olds, suggesting that this gene is downregulated during differentiation states (Figure 3d). We also found a state enriched in *IL23R*, suggesting a population of Tregs within both the 85‐ and < 66‐year‐olds that can respond to IL‐23 cytokine and are amenable to plasticity (Figure 3c,d). Additionally, expression of central memory phenotype (*CCR7*) was also noted in a certain state in Tregs from the 85‐year‐old cohort, along with *RGS1*, thereby highlighting dysfunctional Treg populations in the very old (Flynn et al. 2023). The question remains as to whether the differentiation states can cast some light on the phenotype of Tregs engaged in differential communication with T effectors (Teff). We found that the predominant state that existed in control Tregs was state 1, driven by immune regulatory pathways such as adenosine (state 1 encompassed 32.6% of Tregs enriched in *BTG, CD37*, Figure 3a). In contrast, very old Tregs possessed dual states (17.1%; Figure 3b, Tables S5 and S6). This percentage was derived by calculating the number of Treg cells in the total < 66‐ or 85‐year‐old population to those present in a particular state.

To test the predictive analysis, we performed a flow cytometry analysis. Here, we found that the < 66‐year‐old controls possessed 3 different FoxP3‐expressing populations, while the 85 cohort possessed four different populations (Figure S2a), highlighting Treg heterogeneity in the 85 cohort compared to the < 66 cohort. Hence, pseudotime analysis reveals the existence of several Treg states in the 85‐year‐old individuals, which may resolve the various contradictory studies existing in current literature.

### 2.4 Aged Tregs May Possess Enhanced Crosstalk With CD8 ^+^ CD56 ^+^ KLRB1 ^−^ T Cells

Using CellChat analysis in R software (Jin et al. 2021), we investigated the signaling patterns of immune cells in peripheral blood. CellChat predicts the major signaling inputs and outputs of cells by employing network analysis and pattern recognition approaches to predict how cells and signals coordinate for functions. By leveraging a comprehensive database of interactions among ligands, receptors, and their cofactors that accurately represents known heteromeric molecular complexes, CellChat analysis can distinguish global communications among cells (Jin et al. 2021). Using this analysis, we found that in the 85‐year‐old cohort, five different incoming communication patterns were detected in immune cells, compared to just two in the < 66‐year‐old controls (Figure S3a,b). Specifically, in Tregs, we explored the co‐receptor signaling pathways and found that incoming signals through CTLA4 (CD80/CD86) and CD30 (CD30L) were active in both age groups (Figure S3a,b, denoted with a red box) with the primary difference observed in the IL4 incoming signals [denoted by a blue box in S3b]. We next determined the co‐receptor pathways that aged Tregs predominantly utilized to communicate with other immune cells (outgoing signals) and found that in comparison with Tregs from the < 66 group, 85‐year‐old Tregs exhibited several non‐dominant signals using both the CD80 (CTLA4) and CD30L (Figure S3c,d, denoted with a red box) co‐receptor signaling pathways. Furthermore, Tregs from the 85 group displayed pattern 1 signals that were largely dominated by CD48 and LCK signaling pathways, whereas pattern 6 was enriched in CD80 and CD30L, and pattern 7 was enriched in FASLG signals (Figure S3d). This differed in Tregs within the < 66 cohort, where pattern 1 was enriched in CD80 and CD30L, pattern 2 in CD86 and Galectin, and pattern 4 in CCL (Figure S3c). Hence, in 85‐year‐olds, Tregs may upregulate LCK, a kinase that is involved in transducing T‐cell receptor signaling, suggesting T‐cell receptor signaling may be highly active in Tregs of this age. Tregs contained within Pattern 6 may signal through co‐receptors, and Tregs contained within pattern 7 may signal through FASL.

**FIGURE 4:**
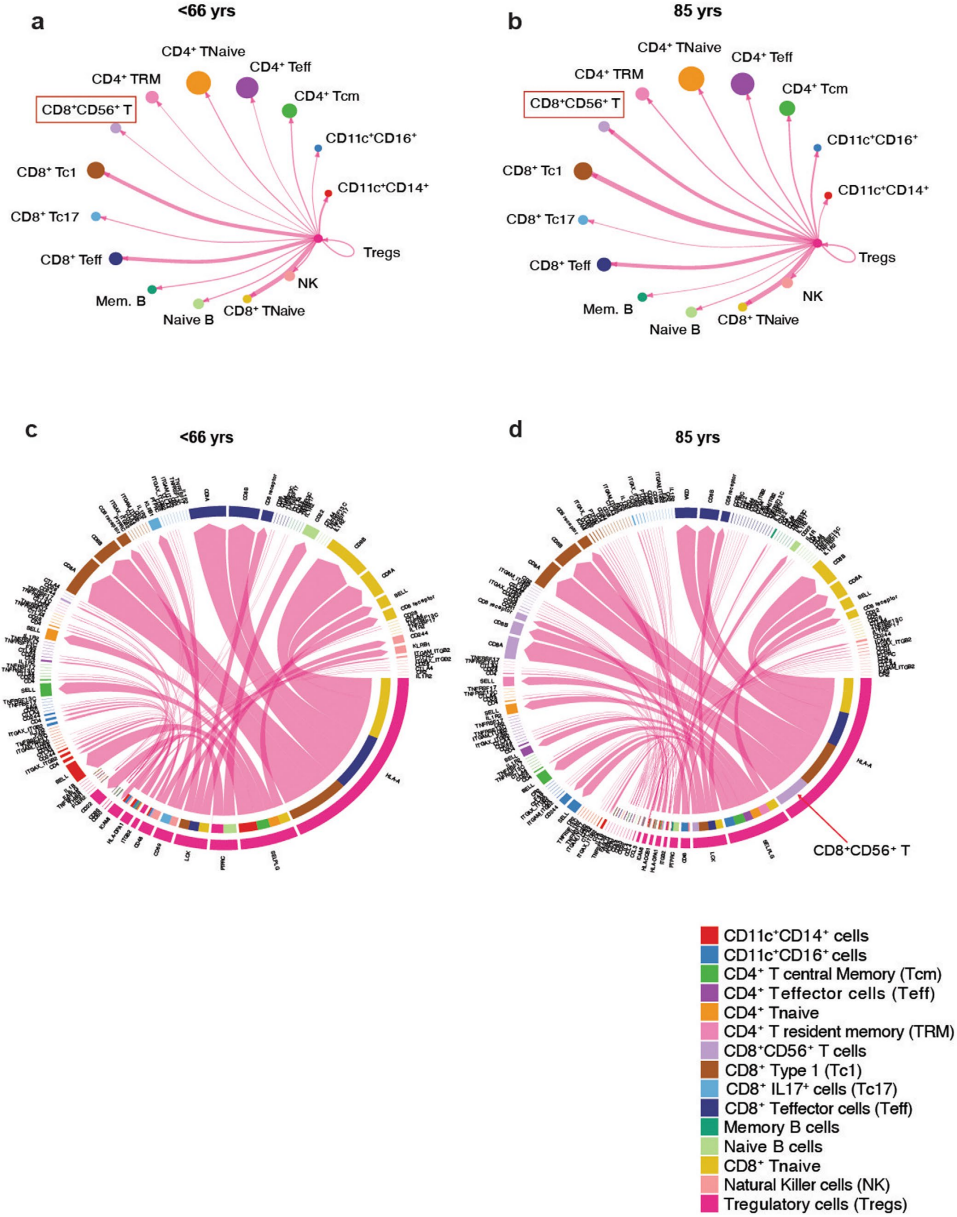
CellChat Analysis identifies upregulation of Treg crosstalk with CD8^+^CD56^+^ T cells in aging. CellChat analysis was carried out using R Studio on the single‐cell data to determine communication pathways in aging. CellChat uses a ligand‐receptor pair database to infer communications between cell types. The outgoing signals from Tregs in < 66‐ (a) and 85‐year‐old (b) are visualized in circle plots. Corresponding chord diagrams of specific ligand‐receptor pair interactions of Tregs with other immune cell populations for < 66‐ (c) and 85‐year‐old (d) are shown, whereby the thickness of the line indicates the strength of interaction, and the number of colored bars indicates the number of interactions per cell type. Tcm, T central memory cells; Teff, T effectors; TRM, resident memory T cells; Tc1, type 1 CD8^+^ T cells; Tc17, IL17 producing CD8^+^ T cells; NK, natural killer cells; Tregs, T regulatory cells; Mem. B, memory B cells.

We next used CellChat analysis to identify the specific populations of immune cells that aged Tregs communicated with. This analysis revealed that Tregs from the very old specifically interact with a population of natural killer‐like CD8^+^ T cells (CD8^+^CD56^+^CD3^+^CD161^−^ or NKT cells), denoted by the thickness of the arrow line. In addition, we noted a slight increase in communication with various T helper (Th) cell subsets, though these were less pronounced than those with NKT cells. NKT cells have been implicated in antitumor responses and in chronic antiviral responses in humans (Satoh et al. 1996). In aging, it appears that Tregs actively interact with this population (Figure 4a,b). Specific interactions also showed an enhanced interaction of Tregs with killer‐like CD8^+^ T cells (Figure 4c,d, see red arrow in d).

### 2.5 Unbiased Analysis of Treg Ligand‐Receptor Pairs That May Interact With Immune Cells in Aging

**FIGURE 5:**
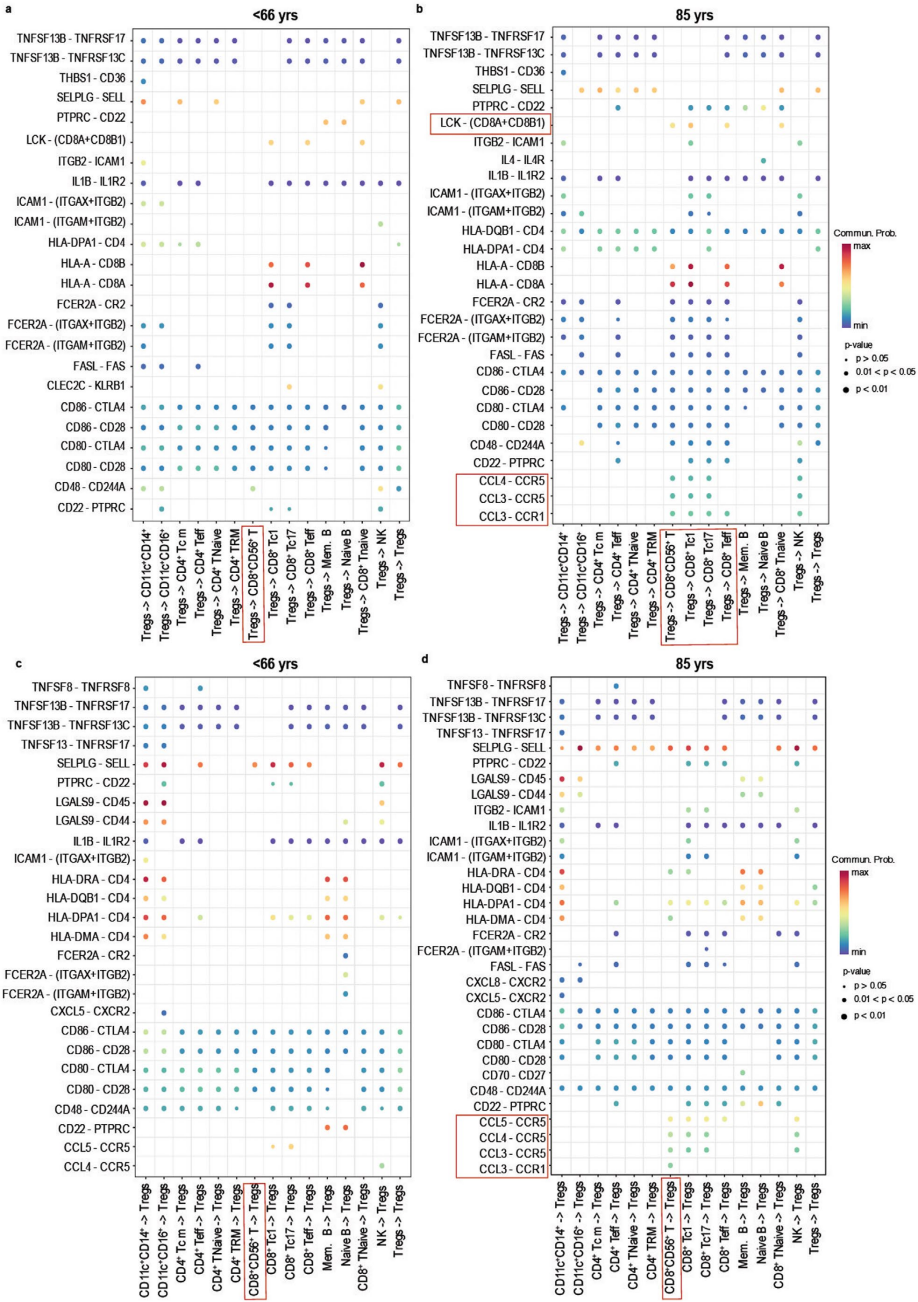
CellChat Analysis identifies altered specific ligand–receptor pairs in aging. On identifying the patterns of communication and Treg signaling pathways, further analysis was performed to understand the ligand‐receptor pair interactions between Tregs and CD8^+^CD56^+^ T cells. Treg ligand or receptor interacting with all immune cells in < 66‐ (a) and 85‐year‐olds (b). Here, Treg interaction with CD8^+^CD56^+^ T cells is highlighted with red boxes. Incoming signals from immune cell populations that are interacting with Tregs in < 66‐ (c) and 85‐year‐olds (d) are shown. Interaction between CD8^+^CD56^+^ T cells and Tregs is highlighted by a red box. Dot color represents communication probability, dot size reflects computed *p* values, and an empty space indicates a zero value of communication probability.

To further interrogate specific interactions, we next performed a ligand‐receptor pair interaction heatmap analysis to determine the crosstalk between Tregs and other immune cell types. Here, we found that in comparison with the < 66 cohort, Tregs within the 85 cohort interact with several immune cell subsets through distinct signaling receptors such as *LCK* and chemokine receptors (Figure 5a–d, denoted by red boxes). Although chemokine signals were not present in the pattern graphs for 85‐year‐old Tregs in Figure S3, it is important to note that these chemokine receptors are expressed by Tregs and are predicted to drive Treg interaction with NKT and CD8^+^ T‐cell subsets, albeit the communication probability is not as strong as *LCK*. This mRNA data suggests that, in aging, Tregs may be enriched in specific interactions with CD8^+^CD56^+^ T cells. Next, we performed an unbiased analysis to determine which non‐Treg immune cell population exhibited enhanced signaling crosstalk in aging. Our findings indicate that the CD8^+^CD56^+^ T‐cell population was the dominant source of “incoming signals” to other immune cells (Figure S4a,b, highlighted by red boxes). Similarly, this CD8^+^CD56^+^ T‐cell population also accounted for the majority of “outgoing signals” (Figure S4c,d, denoted by red boxes). Although minor changes were noted in the CD4^+^ Teff population, this was not as significant as those observed in CD8^+^CD56^+^ T cells.

### 2.6 Aged CD8 ^+^ CD56 ^+^ NKT Cells Do Not Show Enhanced Crosstalk With Tregs

**FIGURE 6:**
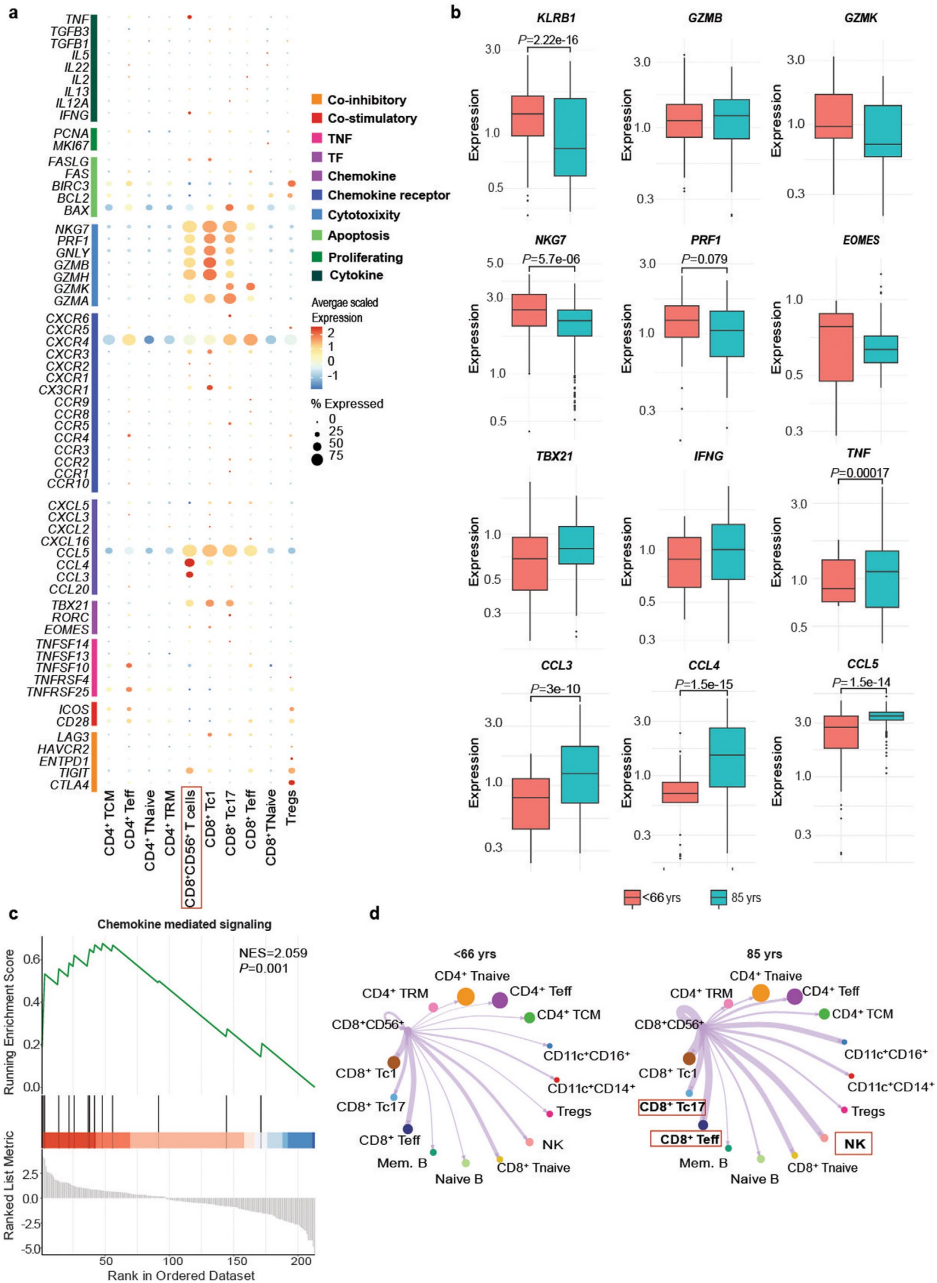
Aged CD8^+^CD56^+^ T cells show an altered chemokine and cytotoxic gene expression profile and altered communication patterns. The CD8^+^CD56^+^ T‐cell subset was isolated from other immune cells, and transcript expression of particular markers is shown (a) in a bubble heatmap. The type 1 phenotype, cytotoxicity, and chemokine expression profile of CD8^+^CD56^+^ T cells were measured (b) and shown in boxplots. Chemokine‐mediated signaling was upregulated in 85‐year‐old CD8^+^CD56^+^ T cells by gene set enrichment analysis (c). CellChat analysis was applied to CD8^+^CD56^+^ T cells to determine the strength of outgoing signals to other immune cell populations in < 66‐ and 85‐year‐old individuals (d), whereby the thickness of the line indicates the strength of the signal. Statistical analysis for the boxplot was determined using the Wilcoxon test. Tcm, T central memory cells; Teff, T effectors; TRM, resident memory T cells; Tc1, type 1 CD8^+^ T cells; Tc17, IL17 producing CD8^+^ T cells; NK, natural killer cells; Tregs, T regulatory cells; Mem. B, memory B cells.

To confirm that CD8^+^CD56^+^ NKT cells were potentially regulated by Tregs and not vice versa, we next determined the immunobiology and crosstalk of aged CD8^+^CD56^+^ T cells. We found that this population was enriched in chemokines and cytotoxicity markers in age (Figure 6a). We then compared the cytotoxicity profile, type 1 phenotype, and chemokine expression of CD8^+^CD56^+^ T cells between < 66‐ and 85‐year‐old individuals. With aging, CD8^+^CD56^+^ T cells tended to possess decreased perforin but not granzyme cytotoxicity, and an increased type 1 phenotype. CD8^+^CD56^+^ T cells from the very old did show a significant increase in *TNF* production and chemokine expression (Figure 6b) along with enhanced immune response pathways (Figure S5a) and chemokine signaling pathways (Figure 6c). We then used CellChat analysis to assess if these cells communicated with Tregs, driving Treg function. We found that the aged CD8^+^CD56^+^ T cells predominantly interacted with other CD8^+^ Teff cells and natural killer (NK) cells (Figure 6d). To identify specific ligand‐receptor pairs utilized by CD8^+^CD56^+^ T cells to interact with Teff cells, we performed a ligand‐receptor pair analysis, which highlighted several binding partners in aged CD8^+^CD56^+^ T cells as compared to < 66‐year‐old controls (Figure S5b). These data suggest that CD8^+^CD56^+^ T cells significantly communicate and interact with other T cells, but not Tregs, in the older immune system [red and orange dots vs. light green dots]. Taken together, our data suggest that aged Tregs exhibit enhanced crosstalk with a specific subset of killer‐like CD8^+^CD56^+^ T cells which, in aging, communicate extensively via chemokine receptor pairs with other immune cell populations.

## 3 Discussion

This study provides a comprehensive single‐cell analysis of T regulatory cell crosstalk in very old adults (aged 85 years). Using both mRNA and protein expression, along with unbiased analysis, we demonstrate that in aging, Tregs may possess enhanced communication with a type of killer‐like CD8^+^CD56^+^ T cells, which extensively interact with T cells and NK cells in age (Figure S6).

In our study, we analyzed the expression of conventional co‐inhibitory receptors in aged Tregs. The role of co‐inhibitory receptors, namely TIGIT, CTLA4, and PD‐1, has been well documented in Treg function and biology. Previous studies have shown that PD‐1 is crucial for Treg stability (Amarnath et al. 2011; Stathopoulou et al. 2018), but in some cases can also mark dysfunctional Tregs (Tan et al. 2021). Similarly, CTLA4 plays a key role in Treg biology, sequestering CD80 and CD86 from dendritic cells (DC) resulting in dampened T‐cell immunity (Qureshi et al. 2011) but can also cause Treg dysfunction (Paterson et al. 2015). Recently, the role of TIGIT in Treg function has been clarified, showing its importance in inhibiting Th1‐ and Th17‐mediated inflammation (Kurtulus et al. 2015). Thus, we sought to determine if these co‐inhibitory receptors had a differential expression pattern in “very old” Tregs, using an 85‐year‐old cohort. We found a significant alteration of the CTLA4 pathways, a significant change in *TIGIT* mRNA expression, and we also found that certain Treg subsets in the very old lacked TIGIT protein expression but had enhanced PD‐1 expression. Taken together, we conclude there is a reorganization of the co‐receptor profile in aged Tregs, both at the mRNA and protein level, which could directly contribute toward altered Treg function.

The use of antibodies against PD‐1, CTLA4, and TIGIT has been tested in clinical trials for treating cancer patients. Recently, these trials have been expanded to the octogenarians, with surprisingly positive results (Ben‐Betzalel et al. 2019). Herein lies the importance of our finding, whereby anti‐PD‐1 therapy could eliminate those Treg populations which lack TIGIT in the very old, thereby enhancing the function of CD8^+^CD56^+^ T cells. Similarly, our CellChat data show that Tregs function through the CTLA4 signaling pathway in the “very old” and hence by deduction, we can postulate that anti‐CTLA4 therapy can efficiently overcome Treg‐mediated dampening of antitumor immunity. Indeed, most clinical trials with these clinical therapeutics are restricted to measuring common immune subtypes, such as CD8^+^ and CD4^+^ T‐cell subsets. Our data suggest that the enhanced response to checkpoint therapies in the very old could be due to the increase in CD8^+^CD56^+^ T cells. Therefore, our data highlights the importance of understanding co‐receptor heterogeneity and applying this knowledge to Treg communication networks to improve outcomes of future clinical trials and vaccination strategies for the very old. Moreover, anti‐TIGIT clinical trials for cancer patients are ongoing, and our data further support the potential benefit of targeting TIGIT in the very old. Anti‐TIGIT therapy may remove Treg‐mediated dampening of Th1 and Th17 antitumor immunity. Taken together, we propose that these three co‐receptors can be fine‐tuned to maximize antitumor and antiviral immunity in the very old.

In line with published studies, we found that Tregs in very old adults were enriched in *BIRC3* mRNA transcript (Raynor et al. 2015) however, an increase in ICOS was not found in our protein expression analysis. Hence, we propose that although very old Tregs have increased *BIRC3* transcript, which may render an anti‐apoptotic phenotype, this was not corroborated with increased ICOS expression in contrast to murine studies (Raynor et al. 2015). We also found that using pseudotime analysis, the differentiation program of Tregs within the 85 cohort was different from that of < 66‐year‐old controls. This feature may explain the contradictory findings on aged Treg function. For example, Tregs that are enriched for *ITGB2* have upregulated superoxide pathways, thereby failing to suppress Tc17 and Tc1 cells, which confirms the ROS study on murine aged Tregs (Guo et al. 2020). At the same time, a subset of Tregs enriched in *RGS1* could render significant suppressive function (Flynn et al. 2023). Ergo, it is important to consider the differentiation states of Treg and other immune cells in very old adults and reconcile functional assays based on the enrichment of specific populations in response to either environmental cues or disease conditions. For instance, a microenvironment driving the expansion of the *RGS1* subset would suggest that enhanced Treg function could occur in that microenvironment in very old adults. Hence, the trajectory and pseudotime analysis identify several differentiation states within very old adults, and it is important to be aware of these various subsets when manipulating the very old immune system. Indeed, it is well established that Tregs upregulate specific transcription factor pathways in order to dampen type 1 (Koch et al. 2009) or type 2 immunity, and the trajectory analysis adds more complexity to this basic biology, whereby specific Treg subsets can be modulated by the oxygenation of the microenvironment, resulting in enhanced or diminished function.

In addition to function, the presence of several Treg differentiation states can also contribute to enhanced crosstalk with other immune cells in the very old. The differentiation states found through the pseudotime analysis were further confirmed by our flow cytometry analysis, whereby we found that Tregs from the very old consisted of four different Treg subsets just based on co‐receptor expression. This heterogeneity expands further when all the mRNA changes are incorporated from the trajectory and pseudotime analysis. Of particular significance, however, is the observation that combining all these changes in the very old Tregs results in enhanced crosstalk with an unconventional immune cell subset, namely a subtype of killer‐like CD8^+^ CD56^+^ T cells, which have been previously implicated in driving antitumor and antiviral responses in aging (Satoh et al. 1996). There is a significant lack of knowledge with respect to the immunobiology of CD8^+^CD56^+^ T cells in health and disease. Our data demonstrate that significant communication links between Tregs and unconventional immune cells need to be further explored in age. The CellChat analysis allows for the identification of key ligand‐receptor pairs that are operational in Tregs within the very old. The importance of co‐receptor signaling was discussed above. In addition to co‐receptors, we also found an increase in signaling through *LCK* [CD8] engagement. This crosstalk was completely absent in the Tregs from the < 66‐year‐old group, suggesting that perhaps an increase in Treg‐specific communication is enhanced with CD8^+^CD56^+^ T cells as they age through either higher expression of CD8 protein, chemokines, or co‐receptors. This increased protein expression of key ligands and receptors could, in turn, increase the affinity of Tregs toward CD8^+^CD56^+^ immune cell subsets. Hence, future studies on aging should include understanding the biology, frequency, and function of unconventional killer‐like T cells in healthy individuals.

Our data demonstrate that Treg function in age is unique and could contribute to the underlying unchecked inflammaging in the very old. The potential of Tregs to control the aberrant function of CD4^+^ and CD8^+^ T cells may be insufficient, since most Treg function is sequestered toward killer‐like CD8^+^ CD56^+^ T cells, and this could drive the persistent inflammation observed in very old adults. Hence, it may be important to determine ways to rewire this communication phenotype of very old Tregs, whereby they control unwarranted inflammation caused by Teff cells. Such re‐channeling of Treg affinity is an area of research that is underappreciated and is yet to be investigated. Taken together, our data is the first to show the complexity of Treg biology and crosstalk in the very old immune system.

Several aging‐related and immune phenotyping studies in the very old have been previously published in the Newcastle 85+ Study (Collerton et al. 2009), a prospective study of bio‐psycho‐social factors that influence the aging trajectory of very old adults. Our study, using participants from the MASS_Pilot (Dodds et al. 2018) provides a deeper understanding of the regulatory immunophenotype of very old individuals using single‐cell analysis. In humans, several single‐cell analyses of the aged immune system have been carried out (Zheng et al. 2020), but the significant drawback of most aging studies is the use of participants who are < 80 years old (Terekhova et al. 2023; Mogilenko et al. 2021). A single study on supercentenarians has been published with *n* = 7 participants on approximately 41,000 cells (Hashimoto et al. 2019). However, a major limitation of this work is the lack of information on how the various immune cells communicate with each other using ligand‐receptor pair analysis, thereby lacking the in‐depth knowledge of the immune system crosstalk in very old adults. Similarly, a single‐cell analysis on 85+ ‐year‐olds (*n* = 6) was performed by Luo et al. (2022), but this study does not provide an in‐depth analysis of Treg‐related immune regulatory networks. While the limitation of our study includes a smaller study sample than some of the other studies, the strength of our work resides in combining 30 chemokine, co‐receptor, and cell surface proteins for expression analysis, coupled with transcript data, followed by ligand‐receptor pair analysis. Hence, by combining flow cytometry profiles and in‐depth BD Rhapsody single‐cell profiling, we have delineated an important, previously unknown role for Tregs in aging. Given the small volumes of blood collected from the participants in the MASS_Pilot, we were unable to perform further functional studies, given both Tregs and killer‐like CD8^+^ CD56^+^ populations are less abundant in blood. Nevertheless, this is the first study to report on Treg biology in the very old. Furthermore, our study has identified a previously unknown Treg crosstalk in very old adults, suggesting that this immune crosstalk is a major driver of immune regulation in very old adults that is discernible even with a small number of donors.

This study serves as a proof‐of‐concept for unraveling the dichotomous immunobiology that is seen in the very old, whereby enhanced Treg function and low‐grade inflammation co‐exist, while important immune responses, such as antitumor and viral responses, are blunted. Using an unbiased approach, we show that the affinity patterns possessed by Tregs may determine their role in age and in disease microenvironments. This is the first study to postulate a novel immune regulatory axis whereby Treg affinity and communication patterns dictate outcomes.

In summary, our work has identified key aspects associated with Treg biology and communication patterns in the very old, and rewiring this interaction can enhance beneficial immune regulation.

## 4 Materials and Methods

### 4.1 Participant Samples

Three individuals included in this study, aged 85 years (born in 1931) and residing in Newcastle and North Tyneside, were recruited through their general practitioner to the MASS_(Muscle Aging Science Study) Pilot in 2016 (Dodds et al. 2018). These were termed the “85” cohort. A health questionnaire assessed self‐reported long‐term conditions from a list of common age‐related diseases. One participant had one long‐term condition, one had 2 long‐term conditions, and the third had 5 long‐term conditions. The following long‐term conditions were self‐reported as diagnosed by a doctor: cardiovascular diseases such as hypertension and angina in two cases, one case of respiratory disease, one case of metabolic disease, and two cases of past diagnosis of cancer. All participants provided written informed consent to protocols approved by the Tyne & Wear South Research Ethics Committee (15/NE/0382). In this study, *n* = 3 healthy control adults recruited through the blood bank, termed as the “< 66” cohort, were used. These were recruited as leukapheresis products from the NHS blood bank under approved ethics. A table with individual information is shown in Table S7.

### 4.2 Cell Staining and Sorting

Single‐cell RNA sequencing was carried out according to the manufacturer's protocol using the BD Rhapsody single‐cell analysis system (BD Biosciences) (Gao et al. 2020). Patient samples were thawed and stained with PE‐conjugated anti‐human CD45 (BioLegend, clone; HI30) for 30 min at 4°C. Samples were then incubated for 20 min with a unique sample tag using the BD Human Single‐Cell Multiplexing Kit (BD Biosciences). Samples were washed in PBS supplemented with 5% FBS, stained with 4′,6‐diamidino‐2‐phenylindole (DAPI), and sorted for CD45^+^ DAPI^−^ cells. Cells were sorted using a BD FACSAria Fusion Cell Sorter. Sorted samples were pooled in equal quantities and incubated with TruStain FcX (Biolegend) and BD AbSeq Immune Discovery Panel antibodies (BD Biosciences; CD3, CD4, CD8, CD11C, CD14, CD16, CD19, CD25, CD27, CD28, CD45RA, CD56, CD62L, CD127, CD134, CD137, CD161, CD183, CD185, CD186, CD196, CCR7, CD272, CD278, CD279, GITR, TIM3, HLA‐DR, IgD, IgM) to detect protein expression. BD Rhapsody cartridges were primed, and the cell suspension was loaded and incubated for 20 min at RT. Cell capture beads with a unique molecular identifier (UMI) and cell barcode were loaded onto the cartridge. Following cell capture bead incubation, the cells were lysed by cell lysis buffer containing DTT. Beads were retrieved, and reverse transcription was performed. During cDNA synthesis, each molecule is tagged with a UMI and cell label. Following exonuclease I treatment, samples were stored at 4°C for ≤ 3 months.

### 4.3 Single‐Cell Library Preparation and Sequencing

Single‐cell library preparation was performed according to the manufacturer's protocol (BD Biosciences) Sequencing was performed on NovaSeq 6000 (Illumina) using an S2 Reagent Kit v1.5 (200 cycles). The read length was 75 bp R1&R2 for 200 cycles for the single‐cell sequencing.

### 4.4 Preprocessing and Dimensionality Reduction

The BD Rhapsody analysis pipeline (https://www.sevenbridges.com/bdgenomics/) on the seven genomics platform was used to process the sc‐RNA‐seq FastQ files. In R software (v.3.3), the Seurat package (v5.0.3) (Hao et al. 2024) was used to further analyze transcriptomic and protein expression data. Cells were filtered for 1,500–50,000 UMIs and 55–200 genes detected per cell. Multiplets were removed from downstream analysis. Following quality control (QC), the frequency of Tregs in our samples was as follows: < 66–1 = 2.4%; < 66–2 = 1.4%; < 66–3 = 1.2%; 85–1 = 2.0%; 85–2 = 1.7%; 85–3 = 2.6%. The expression matrices were then log normalized using the *NormaliseData* function, and the top 2000 variable features were identified. Samples were integrated (*IntegrateData*) using an anchor set and scaled by z‐score conversion (*ScaleData*). Dimensionality reduction (npcs = 50) was performed using principal component analysis (PCA), and the top 20 principal components were used for Uniform Manifold Approximation and Projection (UMAP). A K‐nearest neighbor graph was constructed (k.param = 20, dims = 1:10, annoy.metric = “euclidean”) and Louvain clustering (resolution = 0.8) was performed. Annotation of clusters was achieved through algorithmically defined gene expression using the *FindAllMarkers* functions (method = “wilcoxon”, adjusted by Bonferroni correction) and further identified utilizing AbSeq protein expression. In total ~ 14,000 cells were analyzed.

### 4.5 Secondary Analysis

Differential gene expression (excluding AbSeq antibodies) was determined using the *FindMarkers* function (method = “wilcoxon”, adjusted by Bonferroni correction) of the Seurat R package with default parameters, and the results were illustrated using the R package *ggplot2* (v3.5.0). Upregulated genes were defined with log_2_‐fold‐change values > 1 and downregulated genes by log_2_‐fold‐change values < −1, both using a *p* value cutoff < 0.05. Using differentially expressed genes, gene ontology (GO) overrepresentation analysis was performed using the *clusterProfiler* (v4.10.1) package and *enrichGO* function. Data were shown using a *barplot* with a *p* value cutoff of 0.05. Gene set enrichment analysis (GSEA) was performed using the *gseGO* function based on the default Kolmogorov–Smirnov test, with Benjamini‐Hochberg correction. Pseudotime and cell trajectory analysis were defined using the Monocle2 Plugin on SeqGeq software for < 66‐ and 85‐year‐old Tregs. All genes (excluding AbSeq antibodies) were utilized as parameters.

### 4.6 Cell–Cell Communications and Signaling Pathways

To identify alterations to cell–cell communication patterns and signaling pathways, we used the cell communications CellChat (v2) R package (https://github.com/sqjin/CellChat). CellChat cross‐references a ligand‐receptor interaction database (CellChatDB.human) including secreted signaling, extracellular matrix (ECM)‐ receptor interactions, and cell–cell contact interactions. Differentially expressed ligands and receptors were determined using the *identifyOverExpressedGenes and identifyOverExpressedInteractions* functions utilizing default parameters. Communications with < 3 cells were excluded (*filterCommunication).* The communication probability was calculated using the *computeCommunProbPathway* function, employing a permutation test. Cell–cell communications were aggregated using the agregateNet_function. The *netVisual_circle* function was used to visualize circle plots, *netVisual_chord_gene* for chord diagrams, and netVisual_bubble for ligand‐receptor interactions from source to target cell groups. Network centrality scores were then determined using the *netAnalysis_computeCentrality* function. The outgoing and incoming signals for each cell were quantified using *netAnalysis_signalingRole_heatmap*. To identify global cell communication patterns, the *selectK* function was applied to select the optimal number of communication patterns, followed by the *identifyCommunicationPatterns* function to compute the global communication networks.

### 4.7 Antibodies and Reagents

X‐VIVO 15 media was obtained from LONZA (Berkshire, United Kingdom). All antibodies (unless otherwise stated) were purchased from BioLegend, BD Biosciences, or eBioscience. The Rhapsody cartridge kit, Enhanced cartridge reagent kit, cDNA kit, Human immune response panel, Targeted mRNA & AbSeq kit, AbSeq immune discovery panel CTT LYO, and Human single‐cell Multiplexing kit were purchased from BD Biosciences and were used for single‐cell sequencing methods.

### 4.8 Lymphocyte Preparation for Flow Cytometry

PBMCs were obtained using lymphocyte separation media by density‐gradient centrifugation. Samples were stored by freezing in 90% FBS and 10% DMSO. Samples were thawed and resuspended in X‐VIVO 15 media supplemented with 5% FBS.

### 4.9 Flow Cytometry

PBMCs were stained with LIVE/DEAD fixable blue Dead Cell Stain Kit as per the manufacturer's protocol (Invitrogen). Cells were then washed with PBS and stained with anti‐ CD19 FITC (clone:HIB19), CD14 FITC (clone:B73.1), CD11c FITC (clone:QA17A16), CD11b FITC (clone:ICRF44), CD56 FITC (clone:S‐HCL‐3), CD16 FITC (clone:QA19A47), TIGIT PerCP‐Cy5.5 (clone:A15153G), CD95 PE‐Cy5 (clone:DX2), LAG3 PECy7 (clone:7H2C65), CD27 APC (clone:M‐T271), CD4 AF700 (clone:SK3), PD‐1 APC‐Cy7 (clone:EH12.2H7), CD45RA BV421 (clone:HI100), CD3 BV510 (clone:SK7), TIM3 BV605 (clone:F38‐2E2), CD45RO BV650 (clone:UCHL1), CD25 BV711 (clone:M‐A251), CCR7 BV785 (clone:2‐L1‐A), CD8 BUV395 (clone:RPA‐T8), CD62L BUV660 (clone:DREG‐56), CD28 BUV805 (clone:CD28.2). Cells were washed with PBS supplemented with 0.1% BSA and 0.01% sodium azide. For intracellular (IC) flow cytometry, fixation and permeabilization buffer was utilized (eBioscience). Cells were then stained overnight with FOXP3 PE (clone:206D) and CTLA4 PE‐CF594 (clone:BNI3) antibody. Data were acquired using a BD Fortessa X20 and analyzed using FlowJo software version 10.8.2.

### 4.10 Statistical Analysis

Single‐cell data was analyzed in R Studio. For box plots generated in R studio, the *FetchData* function was used, using default parameters. The Wilcoxon test was used to compare between groups. Comparison values of *p* ≤ 0.05 were considered statistically significant. Data shown in box plots and violin plots represent gene expression values in the respective groups. Statistical analysis for flow cytometry data was performed using an unpaired Student *t* test.

## Author Contributions

T.M. performed experiments, analyzed data, wrote, and edited the manuscript; J.X.L. provided single‐cell code for cell chat and intellectual input in analyzing single‐cell data in R Studio. K.J.S., B.H., G.H., D.M., R.H., and J.C. performed experiments, while A.A.S., D.A.M., and A.G. edited the manuscript. S.A. conceptualized the idea, wrote the manuscript, and supervised the study.

## Conflicts of Interest

The authors declare no conflicts of interest.

## Supporting information


**Figure S1.** The co‐receptor transcript expression landscape of < 66‐ and 85‐year‐old immune cell populations, PBMCs from < 66‐year‐old controls and 85‐year‐old very old individuals were subjected to BD Rhapsody single‐cell analysis. The frequency of FOXP3^+^ cells as a percentage of total cells, in < 66‐ and 85‐year‐old individuals is shown in (a). To further define the co‐receptor expression landscape, co‐inhibitory receptors (b), co‐stimulatory receptors (c), tumor necrosis family (TNFR) receptors (d) and TNFSF (e) transcript expression across all cell populations are shown as violins. Statistical analysis for box plot were done using the Wilcoxon test.

**Figure S2.** The protein co‐receptor expression landscape of < 66‐ and 85‐year‐old CD4^+^ T‐cell populations, PBMCs from < 66‐year‐old controls and 85‐year‐old “very old” individuals were stained and analyzed by multi‐color flow cytometry. CD4^+^ expressing T‐cell populations were separated from other immune cells identified and the mean fluorescent intensity (MFI) of particular markers was calculated and plotted, as a heatmap^+^(a). Data shown are from *n* = 3 samples for < 66‐ and 85‐year‐old individuals.

**Figure S3.** Cell communications pattern analysis identifies differential Treg communication patterns with aging involving co‐receptor signaling, Using CellChat analysis with single‐cell data, the patterns of global outgoing and incoming signals were determined and plotted as heatmaps. The incoming cell communication patterns and specific signaling pathways driving these communications in < 66 (a) and 85 year‐olds (b) is shown. Outgoing signaling patterns and genes for < 66‐year‐old controls and 85‐year‐old (c, d) immune cell populations are shown. Red indicates a higher contribution of a cell cluster or signaling pathway to a pattern.

**Figure S4.** CD8^+^CD56^+^ T cells are a major contributor to incoming and outgoing signals, Heatmap visualization of incoming signaling pathways received across different cell types in < 66‐ (a) and 85‐year‐old (b) individuals. This is also shown for outgoing signaling patterns (c, d). The color represents the relative strength of the signal. The top bar graph reflects the overall communication strength attributed to a particular cell type, and the horizontal axis pillars reflect overall contribution of signaling pathways.

**Figure S5.** Increased immune response pathways and specific ligand‐receptor pair interactions are observed in CD8^+^ CD56^+^ T cells in age. In R Studio CD8^+^CD56^+^ T cells were isolated from other immune cell populations and gene ontology over representation pathway analysis was performed (a), plotted as a barplot. Further analysis was performed using CellChat to understand the contribution of the ligand‐receptor pairs that were disrupted in CD8^+^CD56^+^ T‐cell crosstalk with other immune cells in < 66 and 85 year‐olds (b).

**Figure S6.** Graphical summary, Graphical summary depicting the changes in the co‐receptor landscape (a), migratory potential (b), differentiation program (c) and communication networks (d) in Tregs from the < 66 year old cohort versus the 85 year‐olds. Created in BioRender. Smith, K. (2025) https://BioRender.com/m43m230

**Table S1.** Cell‐type‐specific markers for assignment of cluster identities.
**TABLE S2.** Differential gene expression for 85‐year‐old Tregs including *p* value and Log_2_‐fold change compared to Tregs from the < 66‐year‐old group.
**TABLE S3.** Gene ontology (GO) pathways enriched in Tregs from the 85‐year‐old cohort.
**TABLE S4.** Gene set enrichment analysis (GSEA) of differentially expressed genes in Tregs from the 85‐year‐old cohort.
**TABLE S5.** Details of singular Tregs from the < 66‐year‐old cohort involved in pseudotime trajectory analysis using Monocle.
**TABLE S6.** Details of singular Tregs from the 85‐year‐old cohort involved in pseudotime trajectory analysis using Monocle.
**TABLE S7.** Patient characteristics used in this study.

## Data Availability

All the data are contained within this manuscript. Single‐cell data have been deposited at GEO under accession number GSE280319, and the code is deposited in GitHub at https://github.com/teganmct/humanscRNAseq_Age.
