# Supplementary figures and images for: Deciphering Novel Communication Patterns in T Regulatory Cells From Very Old Adults
Source: Aging Cell. 2025 Mar 18;24(7):e70044. doi: 10.1111/acel.70044 (PMC12266756; doi:10.1111/acel.70044)

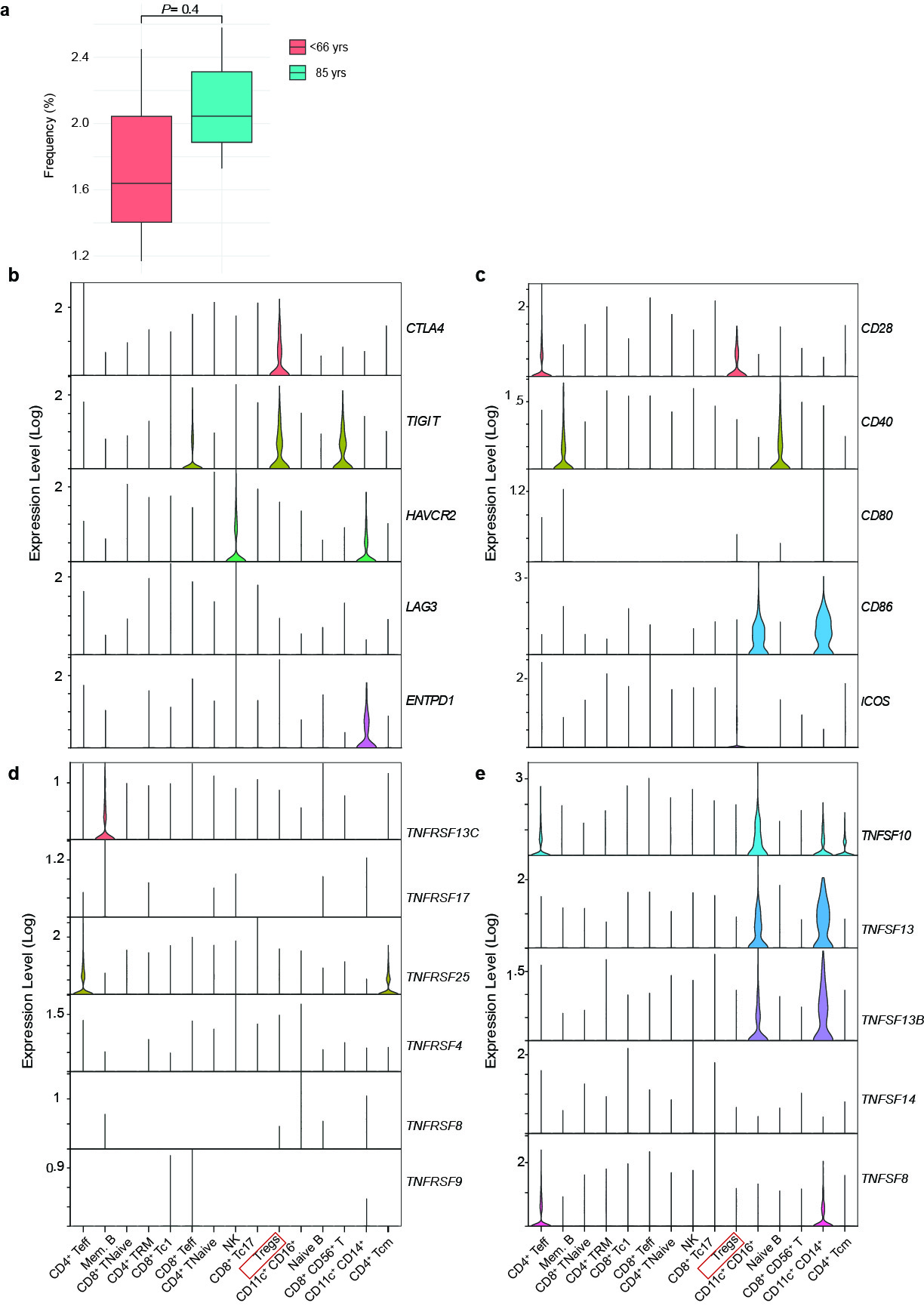

Supplement: Supplementary file 1 — Figure S1. The co‐receptor transcript expression landscape of < 66‐ and 85‐year‐old immune cell populations, PBMCs from < 66‐year‐old controls and 85‐year‐old very old individuals were subjected to BD Rhapsody single‐cell analysis. The frequency of FOXP3+ cells as a percentage of total cells, in < 66‐ and 85‐year‐old individuals is shown in (a). To further define the co‐receptor expression landscape, co‐inhibitory receptors (b), co‐stimulatory receptors (c), tumor necrosis family (TNFR) receptors (d) and TNFSF (e) transcript expression across all cell populations are shown as violins. Statistical analysis for box plot were done using the Wilcoxon test. [file ACEL-24-e70044-s006.jpg]

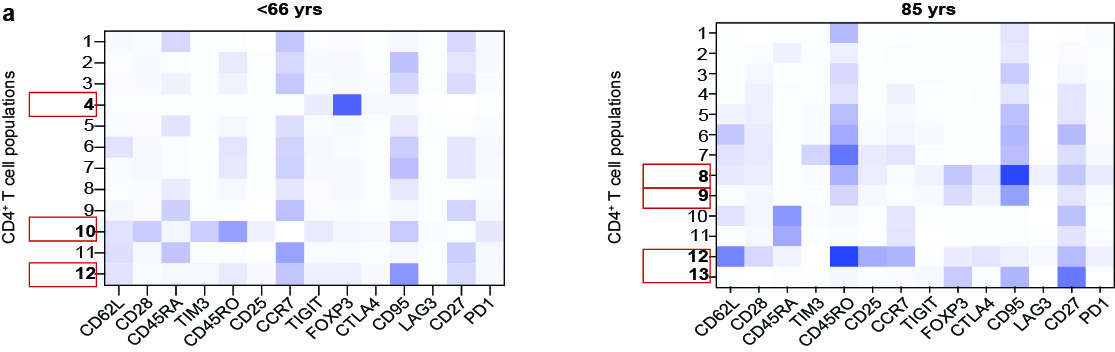

Supplement: Supplementary file 2 — Figure S2. The protein co‐receptor expression landscape of < 66‐ and 85‐year‐old CD4+ T‐cell populations, PBMCs from < 66‐year‐old controls and 85‐year‐old “very old” individuals were stained and analyzed by multi‐color flow cytometry. CD4+ expressing T‐cell populations were separated from other immune cells identified and the mean fluorescent intensity (MFI) of particular markers was calculated and plotted, as a heatmap+(a). Data shown are from n = 3 samples for < 66‐ and 85‐year‐old individuals. [file ACEL-24-e70044-s004.jpg]

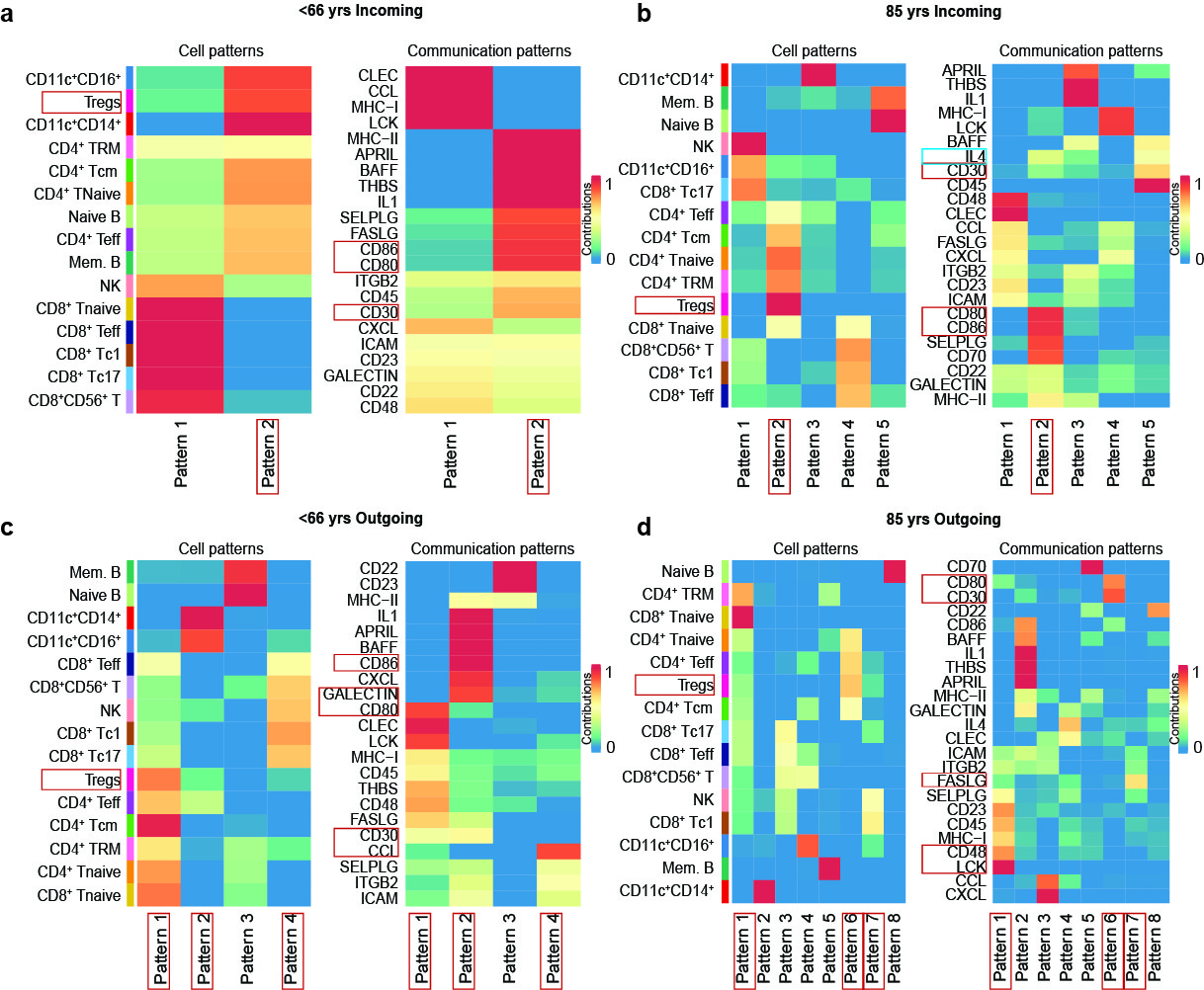

Supplement: Supplementary file 3 — Figure S3. Cell communications pattern analysis identifies differential Treg communication patterns with aging involving co‐receptor signaling, Using CellChat analysis with single‐cell data, the patterns of global outgoing and incoming signals were determined and plotted as heatmaps. The incoming cell communication patterns and specific signaling pathways driving these communications in < 66 (a) and 85 year‐olds (b) is shown. Outgoing signaling patterns and genes for < 66‐year‐old controls and 85‐year‐old (c, d) immune cell populations are shown. Red indicates a higher contribution of a cell cluster or signaling pathway to a pattern. [file ACEL-24-e70044-s002.jpg]

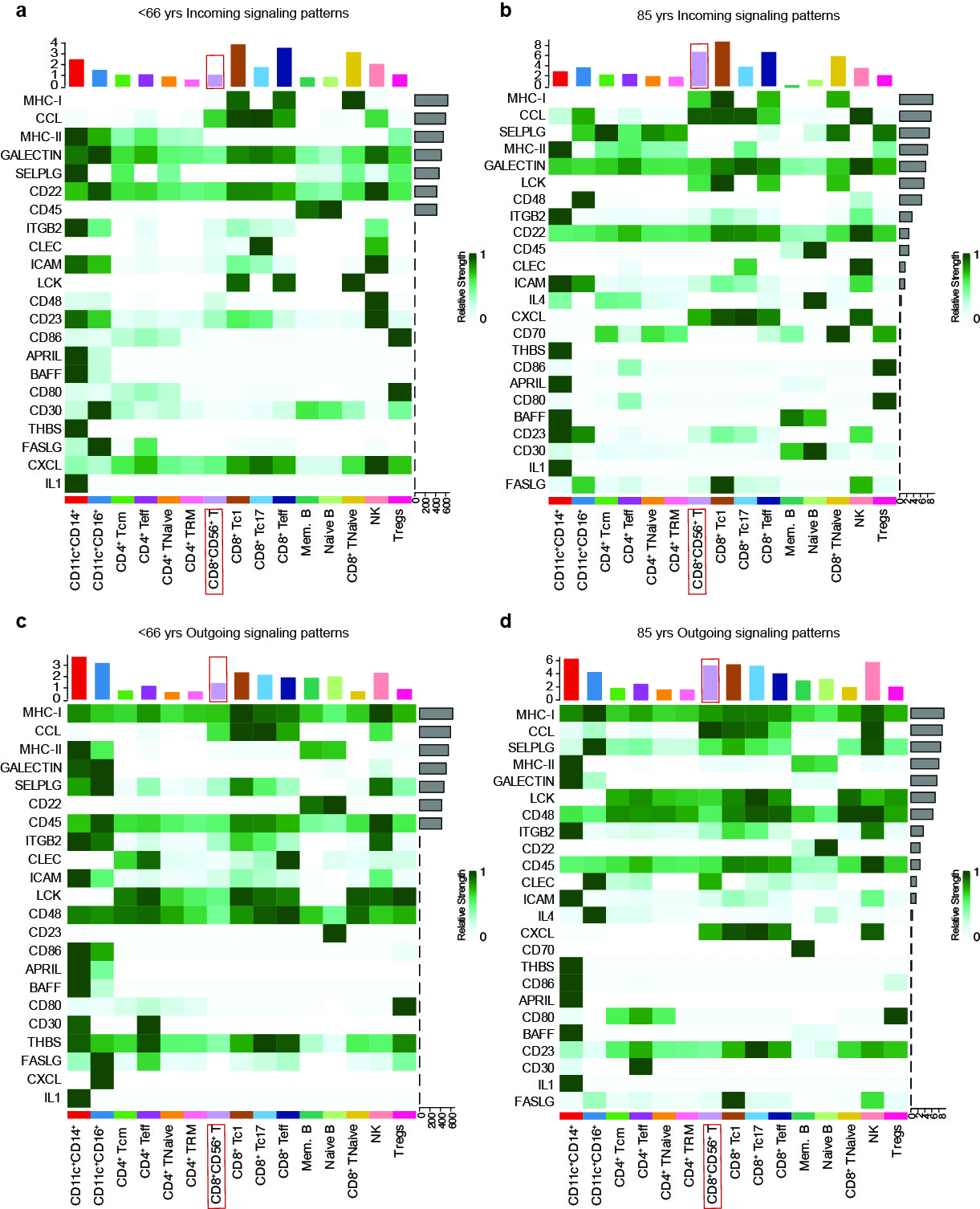

Supplement: Supplementary file 4 — Figure S4. CD8+CD56+ T cells are a major contributor to incoming and outgoing signals, Heatmap visualization of incoming signaling pathways received across different cell types in < 66‐ (a) and 85‐year‐old (b) individuals. This is also shown for outgoing signaling patterns (c, d). The color represents the relative strength of the signal. The top bar graph reflects the overall communication strength attributed to a particular cell type, and the horizontal axis pillars reflect overall contribution of signaling pathways. [file ACEL-24-e70044-s003.jpg]

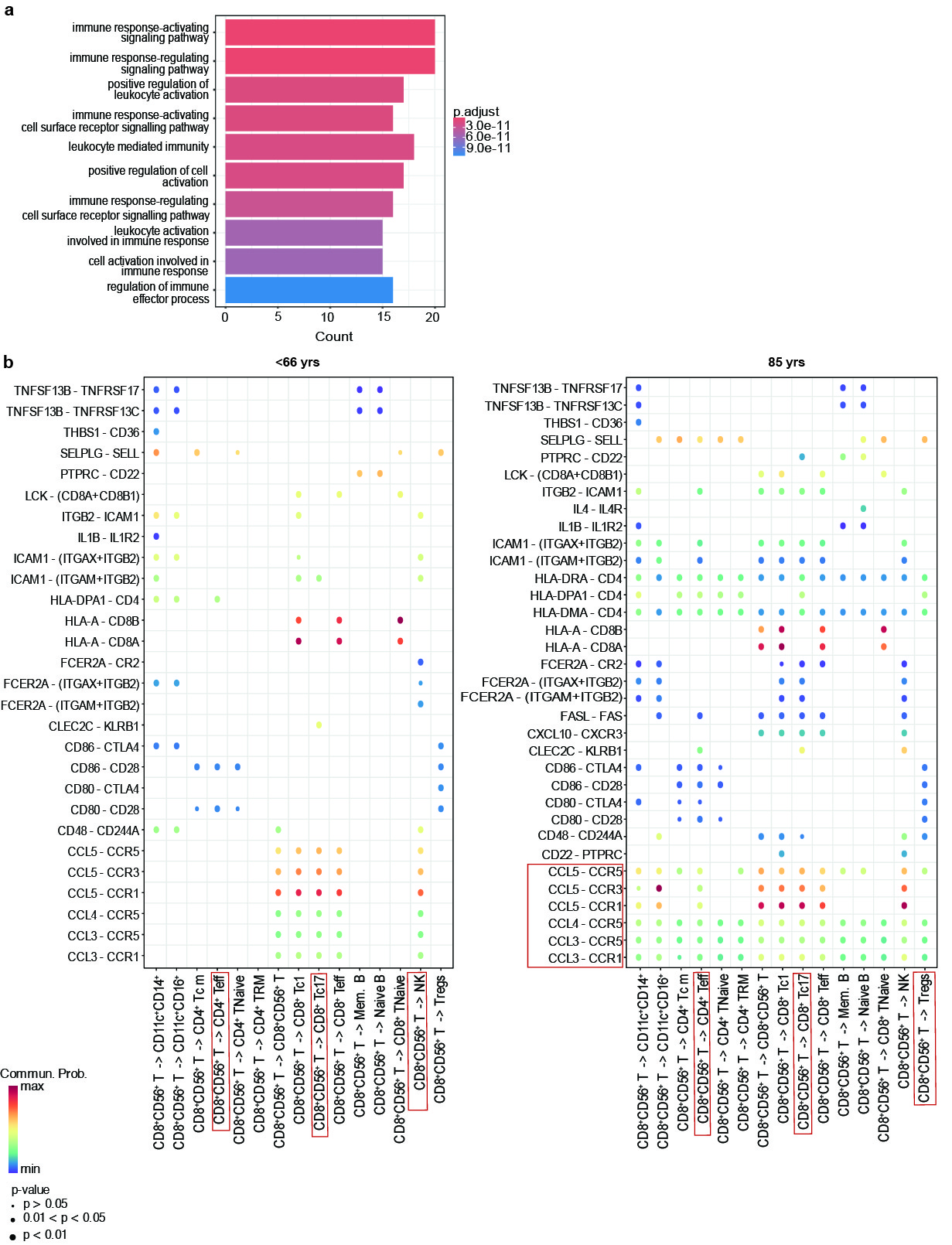

Supplement: Supplementary file 5 — Figure S5. Increased immune response pathways and specific ligand‐receptor pair interactions are observed in CD8+ CD56+ T cells in age. In R Studio CD8+CD56+ T cells were isolated from other immune cell populations and gene ontology over representation pathway analysis was performed (a), plotted as a barplot. Further analysis was performed using CellChat to understand the contribution of the ligand‐receptor pairs that were disrupted in CD8+CD56+ T‐cell crosstalk with other immune cells in < 66 and 85 year‐olds (b). [file ACEL-24-e70044-s005.jpg]

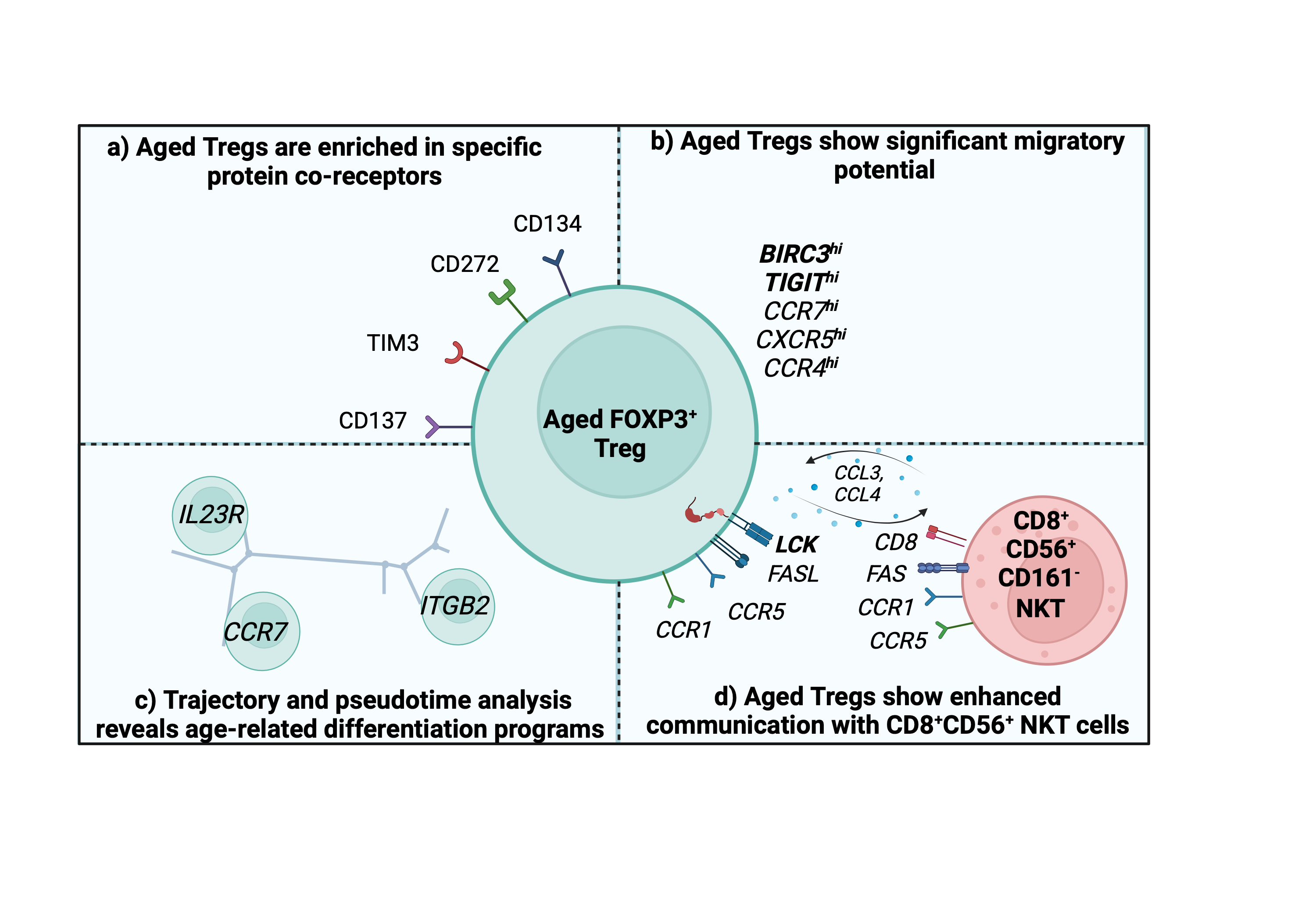

Supplement: Supplementary file 6 — Figure S6. Graphical summary, Graphical summary depicting the changes in the co‐receptor landscape (a), migratory potential (b), differentiation program (c) and communication networks (d) in Tregs from the < 66 year old cohort versus the 85 year‐olds. Created in BioRender. Smith, K. (2025) https://BioRender.com/m43m230 [file ACEL-24-e70044-s007.png]
